# Idiotcy—Its Why and Wherefore

**Published:** 1858-10

**Authors:** 


### IDIOTCY; ITS WHY AND WHEREFORE.*

To whom does the study of idiotcy and its causes belong? To the psychologist? He has laid sole claim to it. To the sanitarian? He has scarcely put forth a finger to touch it. But to which class really does it pertain most? We say to both equally. By the two classes of men it is approached in different directions. The physiologist takes idiotcy as a phenomenon to be studied first by itself, in its details, and as a fact; he must put it in the camera, shed the mental sunlight over it, fix it in its personations, and present it to the world, depictured in all its phases. The sanitarian should take the phenomenon as he is taught it in its details, and thence starting,

* The Causes of Idiotcy; being the Supplement to a Report by Dr. S. G. Howe and the other Commissioners appointed by the Governor of Massachusetts to inquire into the condition of the Idiots of the Commonwealth in 1848.

On Psychological Medicine. By Daniel Noble, M.D. London: Churchill. 1858.

The Human Mind in its relations to the Brain and Nervous System. By Daniel Noble, M.D. London: Churchill. 1858.

The Changes produced in the Nervous System by Civilisation, considered according to the evidence of Physiology and the Philosophy of History. By Dr. Verity. London: 1839.

On the Degeneracy of the Human Race, Physical, Intellectual, and Moral. By Dr. Morel. Paris: 1857.

Twenty-First Annual Report of the Directors of James Murray's Royal Asylum, Perth. Perth: 1858.

proceed from the fact to its causes. We say the duty of the sanitarian *should* be this, because it is a duty which has been neglected. It is scarcely, however, the fault of the sanitarian this neglect; but rather his unavoidable misfortune. His science is new; his co-labourers few; his work laborious; and dealing as yet with more acute disorders,—with epidemics sweeping along in their course, and with diseases of the body which destroy outright. Therefore, has he left the mind and its maladies to his neighbour, but only for a season.

They were still to be lamented the facts we have described, but for the further fact, that the psychologist has not rested at the point where his vocation might seem to cease, but has moved toward the law of causation. Great honour is due to the psychologist for this advancement, to which we willingly award its full praise. The works referred to at the beginning of this paper are all evidences of the debt which the sanitary world owes to the so-called "lunacy doctors". We shall not attempt criticism of these works, for we have not the data for the act; we shall be content to analyze, and by such analysis to lay before our readers what seems nearest in the way of answer to the question, why and wherefore are there a race of men called idiots?

In introducing his researches to the notice of the literary world, Dr. Howe describes how broad a view he and his fellow-commissioners took of the subject before them.

"We did not," he says, "look upon idiotcy as a thing which concerned only the hundred or thousand unfortunate creatures in this generation who are stunted or blighted by it; for even if means could be found of raising all the idiots now within our borders from their brutishness and alleviating their sufferings, the work would have to be done over again, because the next generation would be burdened with an equal number of them. Such means would only cut off the outward cancer, and leave the vicious sources of it in the system. *We regarded idiotcy as a disease of society*; as an *outward sign of an inward malady.* It was hard to believe it to be in the order of Providence that the earth should always be encumbered with so many creatures in the human shape but without the light of human reason. It appeared to us certain that the existence of so many idiots in every generation *must* be the consequence of some violation of the *natural laws*—that where there was so much suffering, there must have been sin. We resolved, therefore, to seek the *sources* of the evil, as well as to gauge the depth and extent of the misery."

"The whole subject of idiotcy is new. Science has not yet thrown her certain light upon its remote or even its proximate causes. There is little doubt, however, that they are to be found in the CONDITION OF THE BODILY ORGANISATION. The size and shape of the head, therefore; the proportionate development of its different parts; the *condition* of the *nervous* system; the *temperament*; the activity of the various functions; the development of the great cavities—the chest and the abdomen; the stature, the weight; every peculiarity, in short, that can be noted in a great number of individuals, may be valuable to future observers."

In the report itself, Dr. Howe observes:

"The moral to be drawn from the existence of the individual idiot is this—he or his parents have so far violated the natural laws, so far marred the beautiful organism of the body, that it is an unfit instrument for the manifestation of the powers of the soul. The moral to be drawn from the prevalent existence of idiotcy in society is that a very large class of persons ignore the conditions upon which alone HEALTH and reason are given to man."

In another place, he gives the following summary:

"Out of 420 cases of congenital idiotcy examined, some information was obtained respecting the condition of the progenitors of 359. Now in all these 359 cases, *save only four*, it is found that one or the other, or both, of the immediate progenitors of the unfortunate sufferers had in some way widely departed from the normal condition of *health*, and violated the natural laws. That is to say, one or the other, or both of them, were *very unhealthy* or scrofulous; or they were hereditarily predisposed to affections of the brain, causing occasional insanity; or they had intermarried with blood relatives; or they had been intemperate, or had been guilty of sensual excesses which impaired their constitutions."

On the subject of *health*, Dr. Howe remarks:

"The health and vigour of the body may be compared to a man's capital; it is a trust fund given to him by the Creator, of which he may expend the interest in the natural enjoyments of life, but he cannot encroach in the least on the principal without real loss. Every debauch, every excess, every undue indulgence, is at the expense of this capital. A rich man may throw away cents or dollars and not feel it, but he is really poorer for it; and a young man with a large capital of health may daily throw away part of it and still feel strong; but every over-stimulant, every overload to the stomach, is a cent or a dollar taken from his capital; feel it or not feel it, he is poorer for it, and so will be the children born to him."

Amongst the five immediate causes of idiocy, Dr. Howe states as the result of his investigations, that by far the most prolific one is "*the low condition of the physical organisation of one or both parents.*"

"It is said by physiologists that among certain classes of miserably paid and poorly fed workmen, the physical system degenerates so rapidly, that the children are feeble and puny, and but few live to maturity; that the grandchildren are still more puny; until in the third or fourth generation, the individuals are no longer able to perpetuate their species, and the ranks must be filled up by fresh subjects from other walks of life, to run the same round of deterioration. It would seem that startled nature having given warning by the degenerated condition of three or four generations, at last refuses to continue a race so monstrous upon the earth."

Once more:

"It will be seen by the tables, that by far the greater part of the idiots are children of parents one or both of whom were of scrofulous temperament and poor flabby organisation. It is difficult to describe exactly the marks which characterise this low organisation, but the eye of a physiologist detects it at once. Regarding it as a matter relating to the mere animal man, if a farmer had swine, cattle, or horses, as inferior to others of their kind as many of these people are inferior to other men and women, he would pronounce them unfit to breed from.

"Great pains have been taken to ascertain the physical peculiarities of the blood relatives of most of the idiots whose names are upon the list. In reading over the description of more than four hundred families in which idiots are found, one is struck with the great number of cases in which *scrofulous* affections are found."

In corroboration of the view that ascribes the largest amount of idiocy to ill health and general deterioration of constitution, we have in the Appendix, No. V, of the work before us, an extract from a *Report on Insanity and Idiotcy in Massachusetts, by the Commission on Lunacy, instituted by the Legislature of* 1854, and drawn up by Dr. Edward Jarvis, who says,

"There is manifestly a much larger ratio of the insane among the poor, and especially among those who are paupers, than among the independent and more prosperous classes. In this connection, it is worth while to look somewhat at the nature of poverty, its origin, and its relation to man and to society. It is usually considered as a single, outward circumstance,—the absence of worldly goods; but this want is a mere incident in this condition, only one of its manifestations. Poverty is an inward principle enrooted deeply within the man, and running through all his elements; it it reaches his body, his health, his intellect, and his moral powers, as well as his estate. In one or other of these elements it may predominate, and in that alone he may seem to be poor; but it usually involves more than one of the elements, often the whole. Hence we find that, among those whom the world calls poor, there is less vital force, a lower tone of life, more ill health, more weakness, more early *death*, a *diminished longevity*. There is also less self-respect, ambition, and hope, more *idiotcy* and *insanity*, and more crime, than among the independent. The preponderance of mental defect and *disease* among the poor is unquestionably shown by the comparison of the number of lunatics and idiots in the two classes."

Dr. Jarvis then shows by statistics, that in Massachusetts, in the year 1854, the ratio of paupers was to the independent as 1 to 47; whereas the lunatics were in the ratio of 72·9 of persons independent to 100 paupers. Hence the pauper class furnishes 64 times as many cases of insanity as the independent class.

In England and Wales (by the *Report of the Commissioners of Lunacy*), it appears that lunacy among the poor was about forty times as great as amongst the independent. The connection between poverty, *ill health*, and lunacy, is well shown in the following remarks of Dr. Jarvis:

"Whatever depreciates the vital energies, lowers the tone of the muscles and diminishes the physical force, and lessens thereby the power of labour and of production, also lowers the tone of the brain and the capacity of self management. In this state, the cerebral organ struggles and may be deranged; consequently we find in the hospital records, that *ill health* is one of the most commonly assigned causes of insanity. It has its first depressing effect on the energy of physical action and the soundness of the judgment in worldly affairs, and next on the power and discipline of the mental faculties."

Dr. Noble, in his interesting and excellent work on *Psychological Medicine*, adopts the same view of the subject. He says,

"The frequency of mental disorder and imbecility amongst the destitute poor, is readily explained by reference to their wretched food and to the miserable *sanitary conditions* to which they are so largely subjected. These circumstances constitute a fruitful source of psychical depravation, as well as of scrofulous degeneracy. Immediately anterior to the breaking out of the dancing mania, there had been throughout many parts of Europe wide-spread wretchedness and misery among large masses of the people."

"Instances of congenital idiotcy and epileptic imbecility result very largely from poor diet and *defective hygiene* generally."

In another part, he refers to the frequency of dwarfed and stunted intellect, as being found in connection with a scrofulous constitution, and cites Dr. Guggenbühl as considering cretinism to consist in something very much allied to scrofula, if not identical with it. In further corroboration, we may state that Mr. Tyerman, medical superintendent of the Colney Hatch Lunatic Asylum, in his *Annual Report* for 1856, in reference to the "Causes of the Disease," says,

"The debility generally observed, and the frequent association of the cases with the insidious affection known as 'general paralysis' and with epilepsy, implies chronicity of *exhausting causes*, concomitants of the hard struggle for a livelihood in this busy metropolis, and in this busy age; an inference confirmed by facts related to me by physicians of the lunatic hospitals of the United States of America, in some of which, general paralytic disease is observed to be gradually extending, and to be keeping pace with competition and the excitement of business among our Trans-atlantic brethren. Some time since I expressed to you my opinion that the disease, as it presents itself in the metropolis, is pre-eminently one of *exhaustion*, and my subsequent observation of its general character has tended more and more to confirm that impression, which is corroborated also by the mode of treatment very generally indicated by the state of the patients and found the most beneficial; a long period of rest, isolation from the excitement of ordinary life, the establishment of regular habits, and the *improved nutrition of the blood*, by a carefully provided *dietary*, being found most important elements in the restoration of its energies to the exhausted brain."

In harmony with the view, that connects idiotcy and insanity with a degeneration of the bodily constitution, Dr. Howe states that,

"In the admissions of the patients to the asylum, the first care has been to put them into the best possible *condition of health and vigour;* to develope strength and activity of body, and to train them to the command and use of muscle and limb."

In connection with this same subject, Dr. Morel's work, *On the Degeneracy of the Human Race, Physical, Intellectual and Moral*, conveys powerful testimony. Dr. Morel points out the degeneracy taking place in Europe from the influence of various causes which act more or less on the constitution and health; amongst these he attributes a large share to *noxious atmosphere*, and states his conviction, that "mental alienation is the last term of a series of degenerations."

"What," he asks, "are in reality the asylums for the insane, but the concentration of the principal degenerations of the human race."

"My actual conviction is, that the insane of our asylums are, in the majority of cases, only the representatives of certain morbid varieties of the race."

"Every where I have heard physicians complaining of the increasing number of the insane, and of the more frequent complications general paralysis, epilepsy, and a more marked depression of the intellectual and physical powers, all of which have contributed to lessen the chances of cure."

"Lastly,—imbecility, congenital, or acquired idiotcy, and other, more or less, complete arrests of development of the faculties of mind or body, indicate in greatly increasing numbers the existence of individuals who receive in fœtal life the principal of degeneracy."

Dr. Morel points out the degenerative influence on the mind and body of the poisonous agencies of marshy effluvia, which he considers the sole cause of the cachexia, known as cretinism; and states his conviction, that in large cities,—

"The same pathologial phenomena are evinced, not only in the acute forms of typhus and typhoid affections, but in the etiolations of the race, and a degradation which yields in nothing to that noticed in marshy districts, and in those affected with cretinism."

Dr. Morel shows, moreover, that London, Liverpool, Manchester, and other large cities, are largely and dangerously charged with these noxious and deteriorating influences, and in the following forcible passage he points out how, by the neglect of due sanitary arrangements, man suicidally manufactures the poison which destroys him.

"The chamber of a person attacked with fever, in an apartment in London, where the fresh air does not circulate, is in a condition perfectly similar to an Ethiopian marsh, where heaps of locusts are rotting. The poison is the same, and only differs in intensity. Nature with her broiling sun, languishing air, and putrid morasses, manufactures pestilence on a large scale; poverty, clad in rags and steeped in filth, excluding the air and increasing the heat, succeeds but too well in imitating Nature."

The view that idiotcy greatly depends on the condition of the constitution; that it is promoted by the deterioration of the system, by impoverishment of the blood, and by the resulting deficiency in nerve force, is insisted on by Dr. Howe, from a fact not, we believe, sufficiently noticed or appreciated; that idiotcy is by no means always, or even generally, accompanied by deficiency in the size, or by deformity in the shape or structure of the head.

Speaking of a very deplorable case of idiotcy in a lad of seventeen. Dr. Howe says,

"His head is not very small, nor is it deformed." But he adds, "the family of which he comes, is very *scrofulous*, and degenerate physically. His relatives (especially his mother) are, many of them remarkable for erysipelatous humours, tumours, carbuncles, etc. One of his cousins is idiotic, though not of so low a degree as he is."

Referring to this case and two others, Dr. Howe further remarks,

"Now, the three persons above mentioned do not seem to be idiotic from any *deficiency* in the *size*, or *deformity* in the *shape* or structure of that part of the *organisation* on which the manifestation of intelligence immediately depends; but there seems to be a want of *power* in that part of the organisation by which the nervous fluid gives energetic action to the frame."

Again, speaking of the measurement of the heads of idiots, Dr. Howe observes,

"It may be stated here, in general terms, that the result of this examination and measurement, shows that no dimensions of the head, except extreme diminutiveness, and no shape whatever can be relied upon as *criteria* of idiotcy. A few of the worst cases of idiotcy, are those in which the head is normal as to size and shape. Nevertheless, the tables show that taking the aggregate of all the cases, an obvious relation is seen between the size and development of the cranium and of its different parts, and the amount of intellectual power, and of the different kinds of mental manifestation." But "because a few persons, with heads below the standard size, have been partially educated, and others with heads of normal size and shape have been hopelessly idiotic, these facts by no means disprove the doctrine of the dependence of mental manifestations upon the structural condition of the brain, because *size* is only *one* of the structural conditions of the brain upon which mental manifestations depend, *quality* of fibre, *health*, exercise, etc., are others essentially modifying it."

This point is further elucidated in the Appendix, No. I, where the importance of *temperament*, or the nature of the tissue of the fibres of the body, especially that of the brain and nervous system, is insisted on.

"The difference of temperament may be recognised by external marks, by the hair, features, skin, proportion of the limbs, and appearance of *fineness* or coarseness in the texture of the body, generally. The perfection of it is best expressed by the single word *blood*, or high blood. There is very great difference amongst men in this respect; the vessel of fine porcelain excels not more in beauty, and especially in fineness of grain, the coarse earthen jug, than does a man of blood or high temperament excel one of low and coarse organisation; no matter, though the first be a North American Indian, the second a prince whose

'Ancient but ignoble blood,
Has crept through scoundrels ever since the flood.'

As the Arabian steed is to the cart-horse, so is the man of fine temperament to the man of coarse temperament."

The opinion here given by Dr. Howe is one at which the physiologist has long since arrived from other, though not less sound, data. We have ourselves expressed in another place to what extent the phrenologists are right, and to what extent they are wrong in their estimate as to organisation and function. We have indicated that in so far as the phrenologist has treated of the conformation of head as one part of the body, he has been right; but that in limiting his observations, on physical development, to the head alone, he has always been wrong. We have shown that a finely balanced brain is unquestionably a grand acquisition; but that like a splendidly built bank minus an imposing capital, the brain is a poor concern without a good chest and stomach to feed it with its full complement of oxygen, nitrogen, phosphorus, water, and other brain-bullion.

Dr. Howe gives two remarkable instances in illustration and proof of this view, in two idiot boys, both with heads below the standard; but the boy with the smallest head, manifesting much more vivacity, activity, and intelligence, than the second, and, indeed, than several of the others.

"Now, why," asks Dr. Howe, "is it that of these boys, whose idiotcy is caused by want of bulk of brain, the one with the *smallest* brain should manifest the most intelligence, and the most character? Precisely for the reason, that the man of 'blood,' or fine temperament, is superior in these respects to the man of coarse organisation, though his brain be smaller; for the same reason that the Arabian steed is superior to the cart-horse, not only in fleetness, but also in sagacity. This boy's body is of a much finer organisation and his brain, doubtless, is so likewise. In his bodily structure, generally, the nervous system has a greater comparative development than in the second, who is rather of a lymphatic temperament. His features are more cleanly cut and chiselled; his skin softer and more delicate, his hair is finer, his eyes are more lively and fiery, his limbs are more delicately shaped, his fingers are longer and differ more from each other in length. As compared with the other, he is more of what may be called the poetic temperament; he is an idiot, but an idiot made of *finer clay*, and in a finer mould." *

Thus the psychologist, proceeding from phenomenon toward cause, has led us by a process of induction which is irresistible in its force, to the primitive fact that idiotcy is neither more nor less than a chronic disease, the origin of which is impoverishment and imperfect nutrition of the body. He has taught us that it is a disease made by man, the sin of the father visited on the child. Henceforth, therefore, it remains to deal with idiotcy as with all evils not ordained but induced; to prevent it; to treat it as a sanitary question; and enforce the precepts announced by Morel, "that physical is inseparable from moral hygiene," and that those "moralists who have need to be convinced that the moral law can only become fruitful in the sound organism", require summary conversion to the faith of physical science.

* This important fact of physiology, as regards the development and progress of the human race, is ably illustrated by Dr. Verity.

